# Supplementary material for: Physical and virtual nutrient flows in global telecoupled agricultural trade networks
Source: Nat Commun. 2023 Apr 26;14:2391. doi: 10.1038/s41467-023-38094-4 (PMC10130181; doi:10.1038/s41467-023-38094-4)
Supplement: Supplementary file 1 — Supplementary Information [file 41467_2023_38094_MOESM1_ESM.pdf]

Supplementary Information for:

Physical and virtual nutrient flows in global telecoupled agricultural trade networks

Xiuzhi Chen[1,2,3,4†], Yue Hou[3,5†], Thomas Kastner[6], Liu Liu[1,2,3], Yuqian Zhang[4], Tuo Yin[3], Mo Li[7], Arunima Malik[8,9], Mengyu Li[9], Kelly R. Thorp[10], Siqi Han[3], Yaoze Liu[11], Tahir Muhammad[3,12], Jianguo Liu[4*] and Yunkai Li[1,2,3*]

1 National Key Laboratory of Efficient Utilization of Agricultural Water Resources, Beijing 100083, China.

2 Engineering Research Center for Agricultural Water-Saving and Water Resources, Ministry of Education, Beijing, China.

3 College of Water Resources and Civil Engineering, China Agricultural University, Beijing, China.

4 Center for Systems Integration and Sustainability, Department of Fisheries and Wildlife, Michigan State University, East Lansing, United States.

5 China International Engineer Consulting Cooperation Overseas Consulting Co., Ltd., Beijing 100048, China.

6 Senckenberg Biodiversity and Climate Research Centre (SBiK-F), Senckenberganlage 25, 60325, Frankfurt-am-Main, Germany

7 School of Humanities and Social Science, The Chinese University of Hong Kong, Shenzhen, Shenzhen 518172, China.

8 ISA, School of Physics A28, The University of Sydney, NSW, Australia.

9 Discipline of Accounting, The University of Sydney Business School, The University of Sydney, NSW, Australia.

10 USDA Agricultural Research Service, 21881 N Cardon Ln., Maricopa, Arizona, United States.

11 Department of Environmental and Sustainable Engineering, University at Albany, State University of New York, 1400 Washington Avenue, Albany, NY 12222, United States.

12 College of Hydrology and Water Resources, Hohai University, Nanjing 210098, China.

[†] The first two authors contributed equally to this work.

* Yunkai Li (yunkai@cau.edu.cn) and Jianguo Liu (liuji@msu.edu) are both corresponding authors.

Section S1. Methods for calculating the unit nutrient contents of agricultural products.

S1.1 Calculation of virtual nutrient contents of agricultural products

S1.1.1 Calculation of virtual nutrient contents of crops

In this study, gridded crop-specific fertilizer, and manure application data at 0.083 degrees, downloaded from EarthStat (http://www.earthstat.org/nutrient-application-major-crops/), were used to extract nitrogen (N) and phosphorus (P) application rates for 17 major crops in more than 221 countries and regions in 2000. These 17 major crops were wheat, maize, rice, barley, millet, sorghum, soybean, sunflower, potato, cassava, sugarcane, sugar beet, oil palm fruit, rapeseed, groundnut, seed cotton and rye. For the calculation of N and P application rates for the other 85 minor crops, as direct data on N and P application rates were not available, crop-specific yield, N and P content, crop-specific harvest area fraction and N and P balance which responded to excess or deficiency of nitrogen and phosphorus compared to the current level were used for the calculation, as shown in the following equation:

$$N_{app} = crop_{yield} \times N_{content} + crop_{HAF} \times crop_{balance} \qquad (1)$$

where N_{app} is the N application rate, kg (see data in Supplementary Data 2); $crop_{yield}$ is the crop-specific yield, kg; $N_{content}$ is the N content; $crop_{HAF}$ is harvested area fraction of the specific crop; $crop_{balance}$ is the N balance of crop, kg, data from EarthStat and (http://www.earthstat.org/total-nutrient-balance-140-crops/) and OECD (https://data.oecd.org/agrland/nutrient-balance.htm).

The calculation process for phosphorus was similar:

$$P_{app} = crop_{yield} \times P_{content} + crop_{HAF} \times crop_{balance} \qquad (2)$$

where P_{app} is the P application rate, kg (see data in Supplementary Data 2); $crop_{yield}$ is the crop-specific yield, kg; $P_{content}$ is the P content; $crop_{HAF}$ is harvested area fraction of the specific crop; $crop_{balance}$ is the P balance of crop, kg, data from EarthStat and (http://www.earthstat.org/total-nutrient-balance-140-crops/) and OECD (https://data.oecd.org/agrland/nutrient-balance.htm).

N and P content data were sourced from https://fao.org/economic/the-statistics-division-ess/publications-studies/publications/nutritive-factors/en/ and https://www.gov.uk/government/publications/composition-of-foods-integrated-dataset-cofid. Crop-specific without N or P content were replaced by taking the average of the same item group. Crop-specific yields and other related data were sourced from http://www.earthstat.org/.

According to literature review and data search, only the year 2000 data is available in the public data at present. Due to the big gap between the existed data and the time range of this study, we used the data of 2000 and combined other available data to estimate the data in 2016 we needed (covering study period from 2000 to 2016). According to principle of total input nutrients consistency, the N and P application rate of 102 different crops in different countries in 2000 were obtained to the analysis. Then, we compared and verified the total nutrient input calculated by the above equilibrium

method with the existing statistical data and found that the difference between the two results was small, which could meet the calculation accuracy (compared the estimated results with nutrients application from FAOSTAT, https://www.fao.org/faostat/en/#data/RFB, the relative error <5%). Finally, we assumed that the changes of nutrients application of different crops were consistent with the changes of the total nutrient application. Based on this rule, the data of 2000 were linearly extended to cover the study period (2000 to 2016).
The N and P contents used in this study were based on the weight of the fruiting part rather than the whole crop-specific yield, which could be uncertain for global-scale calculation. In addition, the N balance deficit of some countries was too large, resulting in negative N and P application rates. In this study, this paper treated it as an outlier and normalized to 0.

S1.1.2 Virtual nutrient calculation of primary livestock products

The virtual nutrients of primary animal products mainly come from feed inputs. We used the feed conversion coefficient to convert virtual nutrients of animals into virtual nutrients of feed crops. The specific formula is as follows:

$$N_i = C_i \times F_i \times N_{feed} \quad (3)$$

$$P_i = C_i \times F_i \times P_{feed} \quad (4)$$

where N_i ,P_i are the virtual N and P content of the animal product, kg t^{-1}; C_i the is the feed conversion coefficient of the animal, kg kg^{-1}, data from Mekonnen & Hoekstra[1]; F_i is the fraction of crop-based feed in total feed, data from Mekonnen & Hoekstra[1]; N_{feed} ,P_{feed} is the virtual N or P content of crop-based feed of the animal product, kg t^{-1}, crop-based feed were partitioned among seven main grain feed commodities - barley, maize, peas, rapeseed, sorghum, soybean, and wheat, data from Herrero et al.[2] All the data for feed conversion coefficients, fractions, and compositions of grains in crop-based feed by animal species and by region are provided in Table S2-S4.

S1.2 Calculation of virtual nutrient contents of processed products

We use factors based on caloric equivalents, according to the method by Kastner[3] to transform the virtual nutrient contents of primary crop or animal products into that of processed products. The calculation formulas are as follows:

$$N_{i,p} = K_i \times N_i \quad (5)$$

$$P_{i,p} = K_i \times P_i \quad (6)$$

where $N_{i,p}, P_{i,p}$ are the virtual N and P content of the processed product, kg t^{-1}; N_i, P_i are the virtual N and P content of the primary product, kg t^{-1}; K_i is the kcal ration between the processed product and the primary product.

The kcal contents of all products are provided in Table S1, according to FAO standard factors on nutritive values (https://www.fao.org/economic/the-statistics-division-

ess/publications-studies/publications/nutritive-factors/en/).

S1.3 Re-export trade data

Most studies have omitted re-export trade flows or have attributed resource use to intermediary trade partners. To account for countries exporting products not grown on their own lands, we followed the Kastner[4] approach to trace crop products back to the most likely producing nation. This approach reverts trade flows to the original producer and final consumer, eliminating intermediate entrepot trade flows. This helps to avoid misattribution of embodied resources[5].

Section S2. Uncertainty analysis and suggestions

Uncertainties in each N or P flow originate mainly from the virtual N or P contents per unit yield of agricultural products with limited data sources for some parameters. These parameters include conversion factors and composition of feed for livestock in different countries. These parameters from current data sources are not distinguished by countries and have not been formally analyzed to determine their uncertainties. Many of the sources of uncertainties are difficult to trace, such as differences between countries in product definitions[5].

Figures

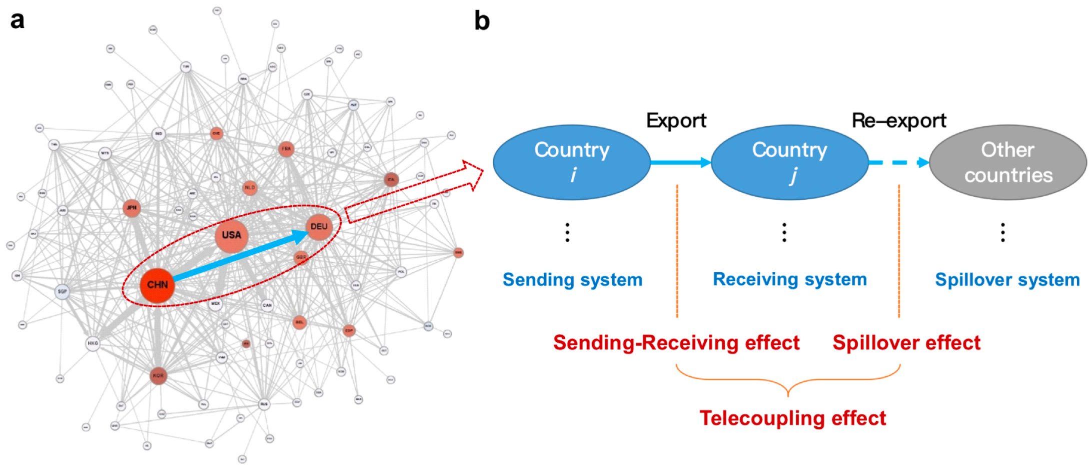

Fig. S1 Systems and effects within the agricultural trade networks under the telecoupling framework. a. Schematic diagram of global agricultural trade networks; b. A specific trade route showing a sending system, receiving system, and spillover system as well as the sending-receiving effect, spillover effect, and telecoupling effect.

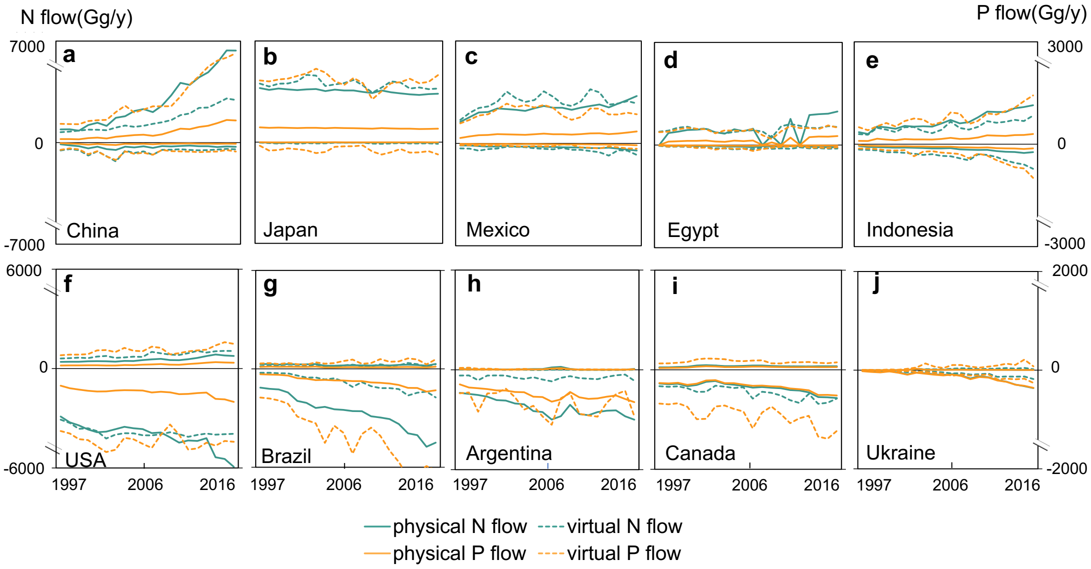

Fig. S2 Changes in nutrient flows over time in major countries | a. China; b. Japan; c. Mexico; d. Egypt; e. Indonesia; f. USA; g. Brazil; h. Argentina; i. Canada; j. Ukraine. Among the top five import and export countries in 2016, many different situations affected the changes in the import and export of nutrients over time. The nutrient imports of China, Egypt and Indonesia showed a fluctuating and increasing trend, while the imports of Japan and Mexico were basically stable, or even declined slightly. The country with the largest increase in physical N and P importing was China, where the increases in N and P received were about six-fold and five-fold, respectively. For exporting countries, the export volume of the United States, Brazil, and Ukraine showed an upward trend in the past 20 years; the nutrient exporting volumes of Brazil and Argentina, the second and the third largest, have both increased by about 4 times. The growth rate of physical N and P exports from the United States is about 90%. Argentina had an upward trend and then a downward trend, reaching its peak in 2007. Brazil's export volume grew rapidly. The export volume in 2016 increased to about 4 times that in 1997. Japan and Indonesia, as large importing countries, had small exports. On the contrary, Argentina and Ukraine, as large exporting countries, had small imports. The changes in the total import and export volume between China and Brazil are related to the soybean import and export trade.

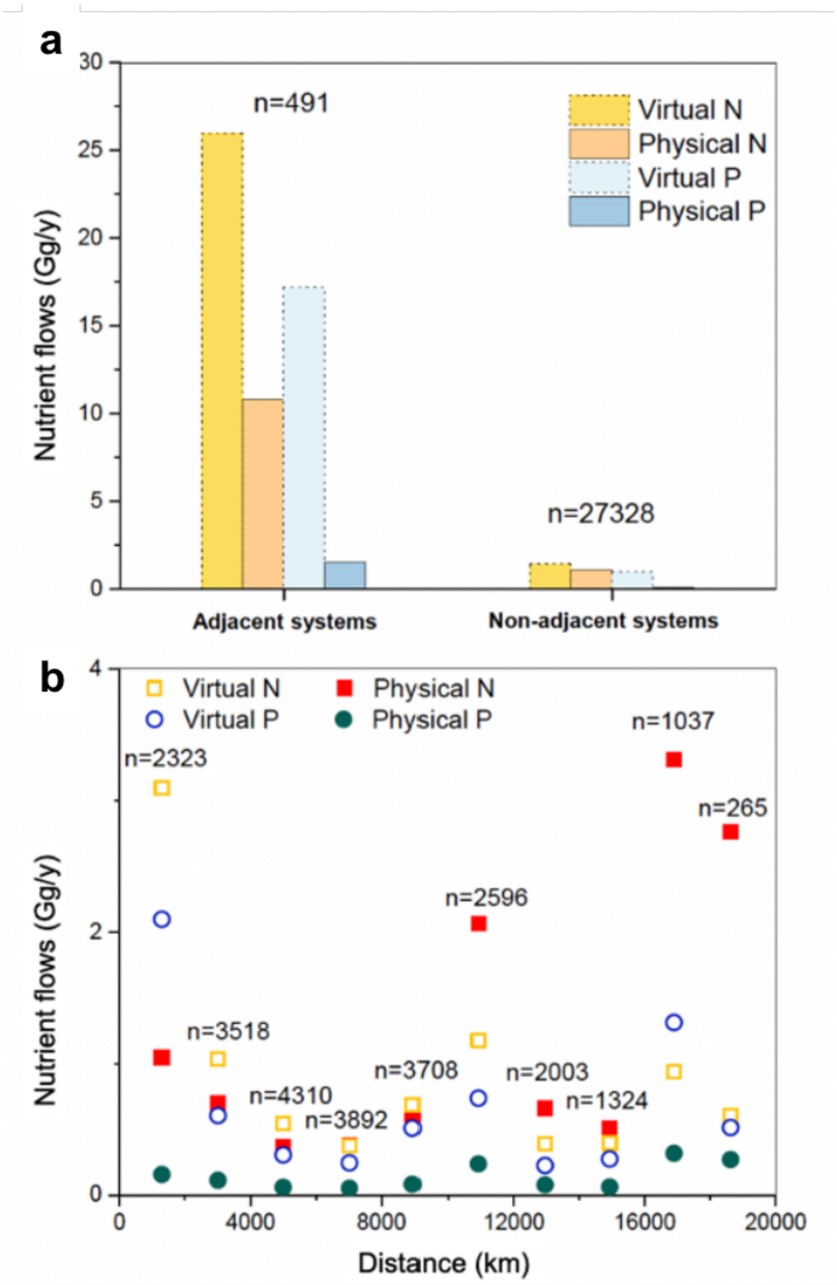

Fig. S3 Average volume of nutrient flow between sending and receiving systems in 2016. a. Average volume of nutrient flow between non-adjacent systems and adjacent systems. b. Average volume of nutrient flow between non-adjacent systems in variable distance. The average flow volume between the adjacent countries was higher than that of non-adjacent countries, which was consistent with a previous finding[7]. When considering only non-adjacent countries at different distances, there is a greater average flow between the countries with greater distances. The border-effect literature notes that most countries or regions trade more with themselves than with any other countries or regions of similar characteristics and sizes[8,9], which may be reflected in our findings.

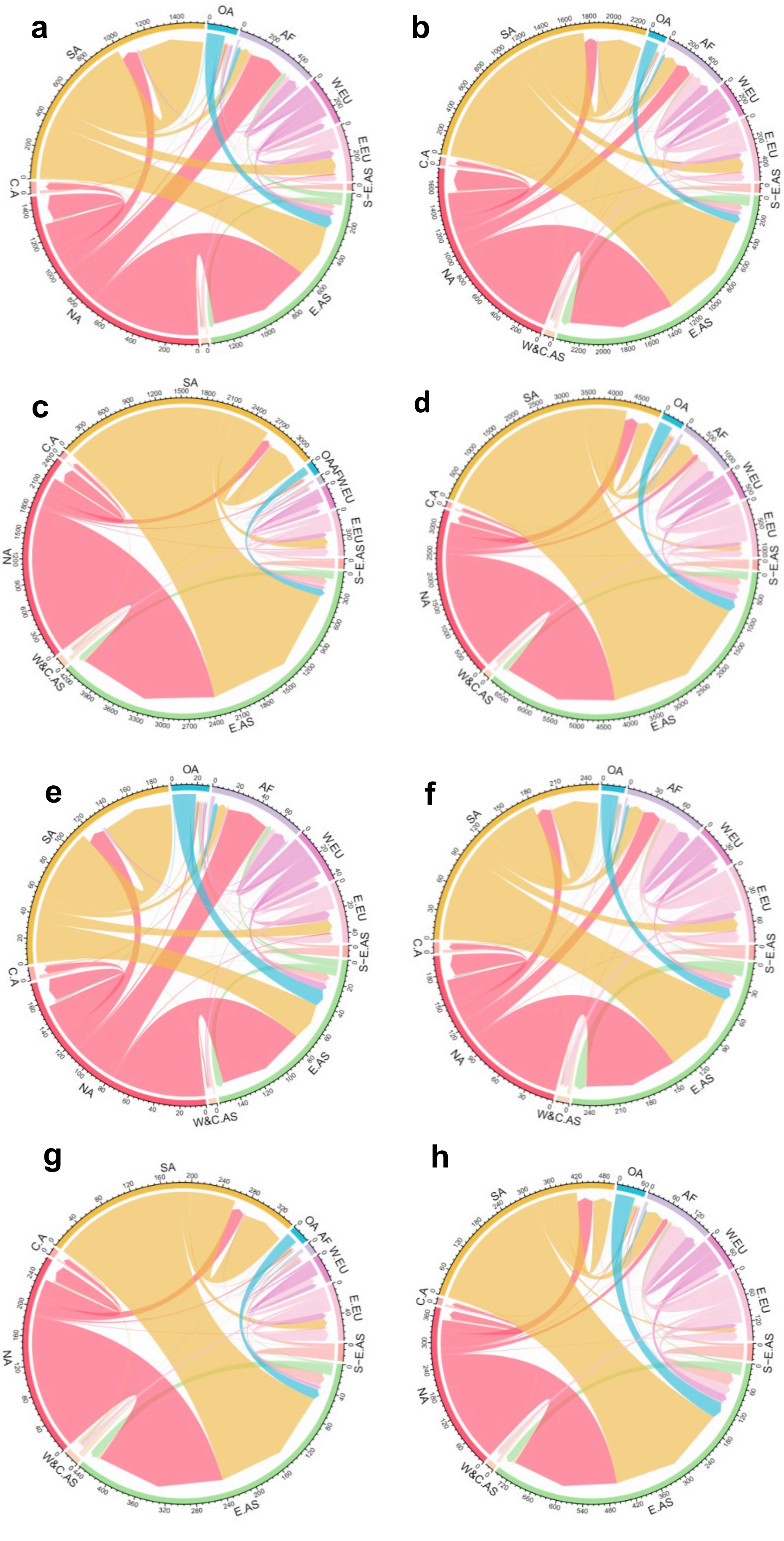
a
b
c
d
e
f
g
h
SA
OA
AF
W.EU
E.EU
S-E.AS
E.AS
W&C.AS
NA
C.A

Fig. S4 Global physical nitrogen (N) and phosphorus (P) flow patterns in 2000, 2005, 2010, and 2015 (Gg y^{-1}). a. Physical N flow pattern in 2000; b. Physical N flow pattern in 2005; c. Physical N flow pattern in 2010; d. Physical N flow pattern in 2015; e. Physical P flow pattern in 2000; f. Physical P flow pattern in 2005; g. Physical P flow pattern in 2010; h. Physical P flow pattern in 2015. The global nutrient flow patterns of physical N and P were almost the same. North America exports to East Asia and South America exports to East Asia were the two largest nutrient flow routes. Southeast Asia was also an important receiving system of nutrient exports from North and South America. In addition, the imported nutrients from Central America also mainly came from North America, while Europe also imported nutrients from South America. Furthermore, the nutrient export volume of the entire European region reached about 20% of the global total in 2015, but it was mainly due to many nutrient flows within the European regions. Oceania almost did not import nutrients, and Africa almost did not export nutrients; and Oceania played a smaller role in the global nutrient flows of agricultural products.

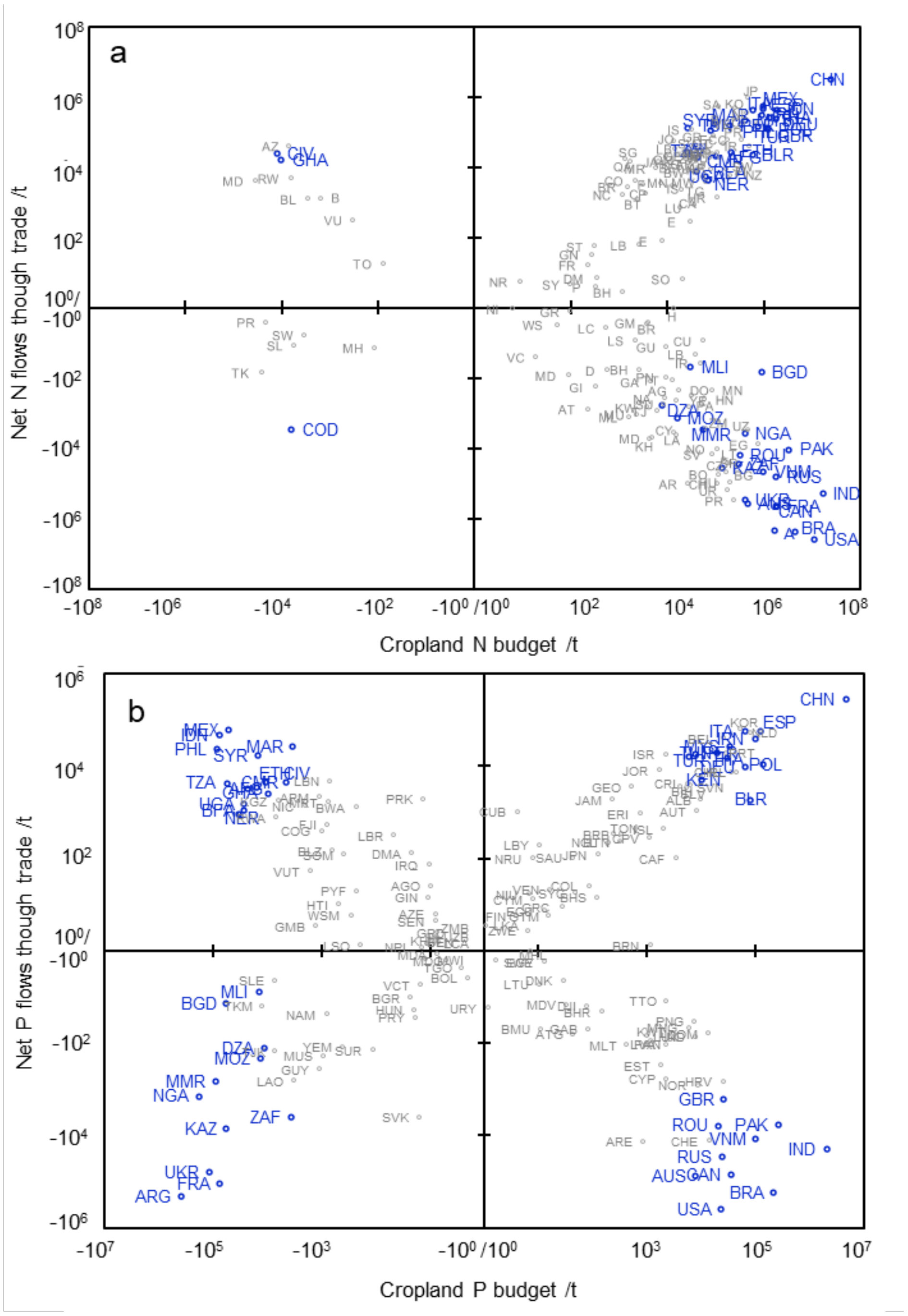

Fig. S5 Cropland budget and net flows though agricultural trade of nitrogen (N) (a) in 2016 and phosphorus (P) (b) in 2016. The country names are abbreviated according to the ISO nomenclature. The colors correspond to the cropland area of the country (blue: ＞50,000 km^2; grey: ≤50,000 km^2. The 48 countries with cropland area greater than 50,000 km^2 account for 87% of all cropland area.) Negative values on the vertical axis represent outflow of N or P, and positive values represent inflow of N or P. Negative values on the horizontal axis represent N or P decrease in local cropland, and positive values represent N or P increase in local cropland.

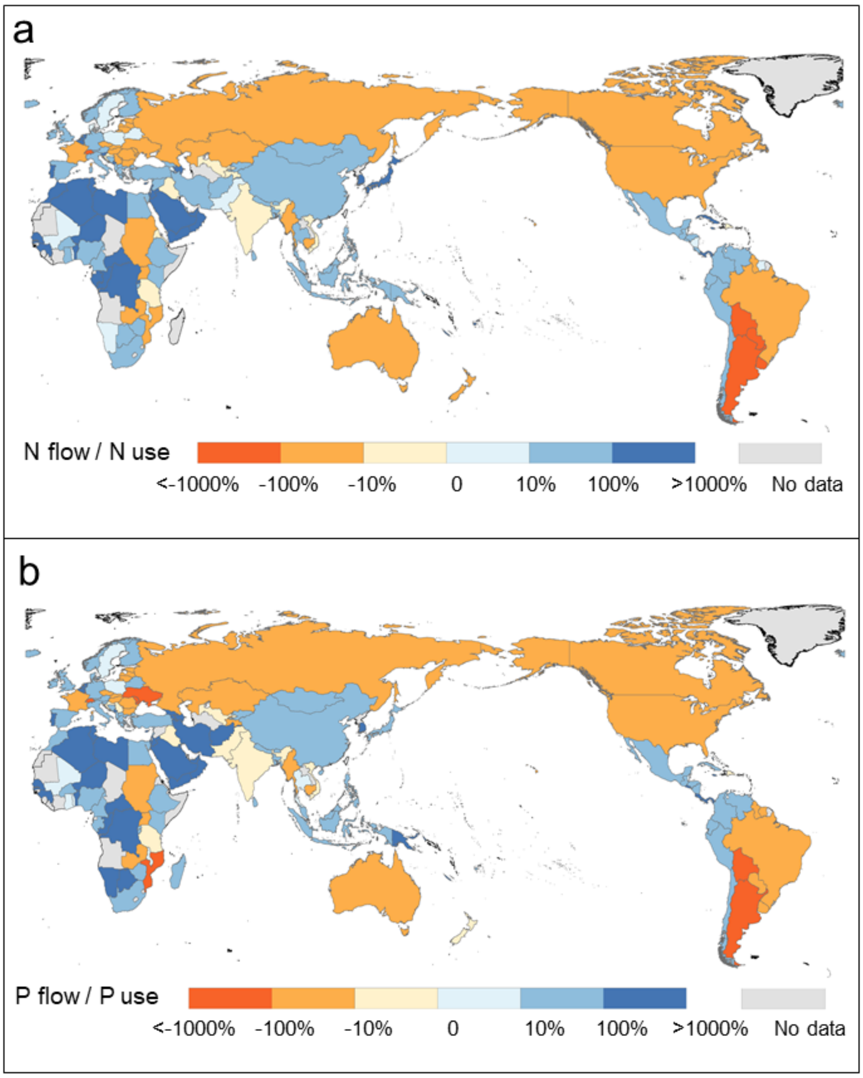

Fig. S6. Physical nitrogen (N) and phosphorus (P) flows through the trade of agricultural products as percentages of domestic mineral N and P fertilizer uses in 2016. a. N flow as a percentage of domestic mineral N; and b. P flow as a percentage of domestic mineral P. Negative values represent outflow of N or P, and positive values represent inflow of N or P. The base map is applied without endorsement from GADM data (https://gadm.org/).

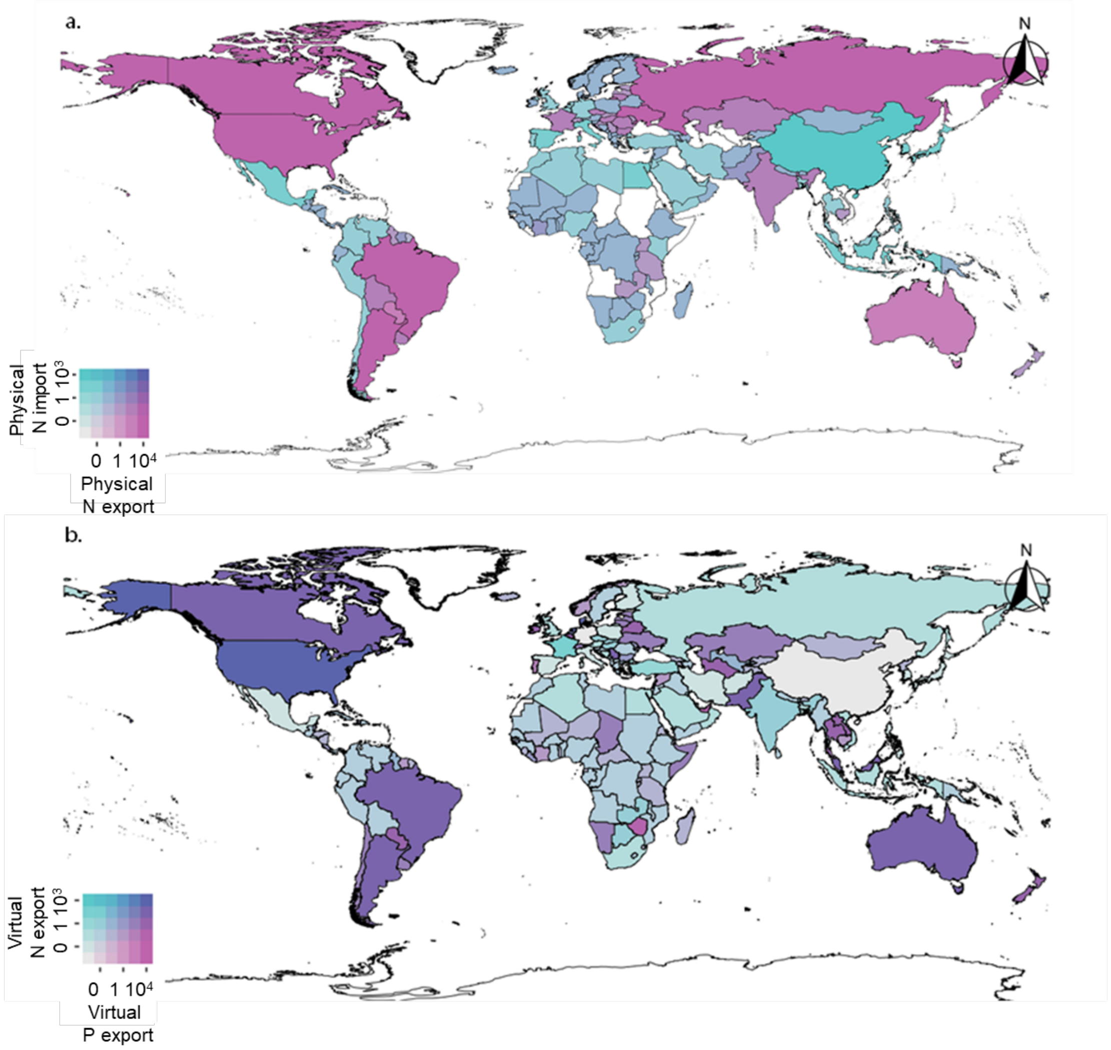

Fig. S7. Comparison of environmental and resource risks by country in 2016. | To identify the dominant characteristics of country's N pollution and P resource problems, we compared the resource and environmental risks caused by import N and export P respectively. Panel a showed the comparison of pollution risk from large import entities N and resource shortage risk from export P; panel b showed the comparison of the risk of pollution borne by massive export of virtual N and the risk of resource shortage caused by export of P. The base map is applied without endorsement from GADM data (https://gadm.org/).

Table S1. Feed conversion coefficient – in kg of feed per kg of output

Animal category	Central and South America	East Asia	East Europe	North America	Oceania	South Asia	Southeast Asia	Sub-Saharan Africa	West Asia and North Africa	West Europe
Beef cattle	56	55	32	25	36	108	71	72	54	28
Dairy cattle	3.2	2.0	1.7	1.1	1.4	2.2	3.7	3.8	2.0	1.5
Broiler chickens	4.5	4.2	4.7	3.4	3.4	5.8	4.7	4.9	4.8	3.6
Layer chickens	3.3	3.1	3.5	2.3	1.9	3.6	3.2	4.0	3.4	2.3
Pigs	6.1	6.4	5.7	4.1	5.3	5.8	6.1	7.0	6.2	5.2
Sheep and goats	72.5	24.8	62.1	30.3	34.2	21.8	18.4	30.2	21.2	33.1

(Note: Data from Mekonnen & Hoekstra[1])

Table S2. Fraction of crop-based feed in total feed dry matter

Animal category	Central and South America	East Asia	East Europe	North America	Oceania	South Asia	Southeast Asia	Sub-Saharan Africa	West Asia and North Africa	West Europe
Beef cattle	0.019	0.035	0.078	0.145	0.011	0.021	0.090	0.045	0.056	0.089
Dairy cattle	0.092	0.366	0.365	0.385	0.099	0.243	0.186	0.083	0.369	0.338
Broiler chickens	0.607	0.766	0.782	0.582	0.596	0.977	0.830	0.833	0.962	0.820
Layer chickens	0.642	0.766	0.789	0.582	0.596	0.977	0.830	0.898	0.961	0.805
Pigs	0.449	0.383	0.758	0.933	0.384	0.349	0.415	0.481	0.838	0.870
Sheep and Goats	0.006	0.002	0.009	0.006	0.002	0.017	0.024	0.019	0.051	0.004

(Note: Data from Mekonnen & Hoekstra[1])

Table S3. Percentage inclusion of grain in crop-based feed for animals [a]

Animal category	Grain category	Europe	US	Brazil	China	LAM[b]	Others
Cattle	barley	0.1	0	0	0	0	0
	maize	0.3	0.5	0.65	0.6	0.6	0.5
	peas	0	0	0	0	0	0
	rice	0	0	0	0	0	0.1
	rapeseed	0.05	0.05	0	0.05	0	0
	sorghum	0	0	0	0	0.05	0.1
	soybean	0.35	0.35	0.3	0.25	0.3	0.2
	wheat	0.2	0.1	0.05	0.1	0.05	0.1
Poultry	barley	0.1	0	0.05	0	0	0.05
	maize	0.25	0.6	0.65	0.7	0.65	0.45
	peas	0.05	0	0	0	0	0
	rice	0	0	0	0.05	0	0.05
	rapeseed	0	0.05	0	0	0	0
	sorghum	0.05	0	0.05	0	0.1	0.15
	soybean	0.2	0.25	0.2	0.2	0.2	0.2
	wheat	0.35	0.1	0.05	0.05	0.05	0.1
Pigs	barley	0.1	0.05	0	0	0	0
	maize	0.15	0.6	0.65	0.65	0.6	0.5
	peas	0.12	0.03	0	0	0	0
	rice	0	0	0	0.05	0	0.05
	rapeseed	0.08	0.05	0	0	0	0
	sorghum	0.1	0	0.05	0	0.15	0.15
	soybean	0.15	0.15	0.2	0.2	0.15	0.15
	wheat	0.3	0.12	0.1	0.1	0.1	0.15
Sheep and Goats	barley	0	0	0	0	0	0
	maize	0.63	0.63	0.63	0.63	0.63	0.63
	peas	0.13	0.13	0.13	0.13	0.13	0.13
	rice	0	0	0	0	0	0
	rapeseed	0	0	0	0	0	0
	sorghum	0	0	0	0	0	0
	soybean	0.14	0.14	0.14	0.14	0.14	0.14
	wheat	0.1	0.1	0.1	0.1	0.1	0.1

(Note: a. Data from Herrero et al [2] b. LAM: Latin America (excluding Brazil))

Table S4. Classification order of agricultural products

Classification	Products	Order	Classification	Products	Order
	Wheat	1.1		Soybeans	2.1
	Maize	1.2		Peas, dry	2.2
	Barley	1.3		Beans, dry	2.3
	Sorghum	1.4		Lentils	2.4
	Oats	1.5		Groundnuts, with shell	2.5
Cereal	Rice, paddy	1.6	Beans & Nuts	Chickpeas	2.6
	Rye	1.7		Broad beans, horse beans, dry	2.7
	Triticale	1.8		Cashew nuts, with shell	2.8
	Millet	1.9		Pistachios	2.9
	Other cereal	1.10		Other beans & nuts	2.10
	Milk, whole fresh cow	3.1		Pigs	4.1
	Cheese, whole cow milk	3.2		Sheep	4.2
	Milk, skimmed dried	3.3		Meat, Chicken	4.3
	Milk, whole dried	3.4		Cattle	4.4
	Whey, dry	3.5		Meat, pig	4.5
Eggs & Dairy	Eggs, hen, in shell	3.6	Meat	Meat, cattle, boneless (beef & veal)	4.6
	Butter, cow milk	3.7		Meat, pork	4.7
	Yoghurt, concentrated or not	3.8		Goats	4.8
	Milk, skimmed cow	3.9		Meal, meat	4.9
	Other eggs & dairy	3.10		Other meat products	4.10
	Oil palm fruit	5.1		Bananas	6.1
	Sugar beet	5.2		Potatoes	6.2
	Rape seed	5.3		Cassava	6.3
	Sunflower seed	5.4		Apples	6.4
	Sesame seed	5.5		Tomatoes	6.5
Sugar & Oils	Linseed	5.6	Veggie & Fruits	Oranges	6.6
	Seed cotton	5.7		Grapes	6.7
	Coconuts	5.8		Watermelons	6.8
	Mustard seed	5.9		Chilies and peppers, green	6.9
	Other sugar & oil crops	5.10		Other veggie & fruits	6.10
Others	Other animal products	7.1			
	Other crop products	7.2			

Supplementary References

1. Mekonnen, M. M. & Hoekstra, A. Y. A Global Assessment of the Water Footprint of Farm Animal Products. *Ecosystems*. **15**, 401-415 (2012).
2. Herrero, M. et al. Biomass use, production, feed efficiencies, and greenhouse gas emissions from global livestock systems. *Proceedings of the National Academy of Sciences*. **110**, 20888 (2013).
3. Kastner, T., Kastner, M. & Nonhebel, S. Tracing distant environmental impacts of agricultural products from a consumer perspective. *Ecological Economics*. **70**, 1032-1040 (2011).
4. Kastner, T., Kastner, M. & Nonhebel, S. Tracing distant environmental impacts of agricultural products from a consumer perspective. *Ecological Economics*. **70**, 1032-1040 (2011).
5. MacDonald, G. K. et al. Rethinking Agricultural Trade Relationships in an Era of Globalization. *Bioscience*. **65**, 275-289 (2015).
6. Lun, F. et al. Global and regional phosphorus budgets in agricultural systems and their implications for phosphorus-use efficiency. *Earth Syst. Sci. Data*. **10**, 1-18 (2018).
7. Xu, Z. et al. Evolution of multiple global virtual material flows. *Science of the Total Environment*. **658**, 659-668 (2019).
8. Chen, N. Intra-national versus international trade in the European Union: why do national borders matter? *Journal of International Economics*. **63**, 93-118 (2004).
9. Okubo, T. The border effect in the Japanese market: A Gravity Model analysis. *Journal of the Japanese and International Economies*. **18**, 1-11 (2004).
