# Supplementary material for: Physical and virtual nutrient flows in global telecoupled agricultural trade networks
Source: Nat Commun. 2023 Apr 26;14:2391. doi: 10.1038/s41467-023-38094-4 (PMC10130181; doi:10.1038/s41467-023-38094-4)
Supplement: Supplementary file 3 — Description of Additional Supplementary Files [file 41467_2023_38094_MOESM3_ESM.pdf]

Description of Additional Supplementary Files:

Supplementary Dataset 1: This dataset contains the list of the 320 products in 8 category considered in the analyses. Physical N,P contents and kcal content used in the calculation are reported for each product.

Supplementary Dataset 2: This dataset contains fertiliser and manure application rates of N and P to soils by crop and by country (2016). The units of value are kg ha-1.

Supplementary Dataset 3: This dataset contains total imports and exports volume of nitrogen and phosphorus, both physical and virtual flows, for each country from 1997 to 2016. The units of value are kg.

Supplementary Dataset 4: This dataset contains IOS3 code for each country or region covered in Table 1.
